# De Morbis quibusdam Commentarii

**Published:** 1782

**Authors:** 


VII. *De Morbis quibusdam Commentarii, auctore* Clifton Wintringham, *Baronetto*, M. D. *Colleg. Medic. Londinens. et Parisiens. Socio, Societatis Regiæ Sodali, et Medico Regio.* 8vo. Cadell, *London*, 1782, 220 pages. 4s.

THIS work, which is offered to the public as the result of forty years experience in military hospitals and in London, consists of 419 aphorisms, to which the author has thought fit to give the name of *Commentarii.*

The following quotation may serve as a specimen of his manner:

90. "Qui semel morbo attonito correptus sit, et postea fiat vertigini obnoxius, novos insultus expectare debet.

91. Qui a paralysi liberantur, punctiones quasi aciculis, uno alterove die in parte affectâ sentiunt, priusquam ad sanitatem perveniant.

92. Exercitationes vehementiores statim post assumptum cibum articulorum morbos gignunt.

93. Si liber sanguinis aut humorum cursus per partem aliquam corporis humani adeo impediatur, ut ex hac causâ aut vires vitales aut animales aut naturales lesæ et oppressæ fiant, neque proprio suo munere fungi queant, cavendum est ne balnei quilibet usus tentetur.

94. Quibus ſit pulſus arteriarum debilis, aut quibus in viſceribus inhæreant ulcera, hic valdè periculoſa ſunt balnea.

95. Dolores poſteriorem capitis partem aut verticem occupantes, plerumque affectioni hyſtericæ indolis tribuendi ſunt.

96. Aquæ minerales morbis febre comitatis exhibendæ non ſunt."

The theory diſplayed in this work is ſuch as might be expected from a diſciple of the Boerhaavian ſchool, and accordingly we read much of *pituita*, *gluten*, acid and alkaline acrimony, and obſtruction as cauſes of diſeaſe. The methods of cure are very often ſuch as we cannot readily adopt. In the Hydrophobia, for inſtance, which is now pretty generally conſidered as a ſpaſmodic diſeaſe, the only remedies preſcribed are fomentations to the *præcordia*, clyſters, a bolus of nitre and ſpermaceti every two or three hours, and repeated bleeding *ad deliquium*. Si autem—ſays the author—ad tertium uſque diem obtineat malum, inſanabile, plerumque erit; tentari tamen poteſt curatio iiſdem pergendo, et repetendo ſanguinis miſſionem ex arteriâ, admotis eodem tempore veſicatoriis, ſi vacillare videatur pulſus."

The cuſtom of drawing the breaſts of lying-in women, women, as well as that of applying ſpirituous embrocations, or plaiſters with the view of repelling the milk, have lately been deſervedly condemned in a judicious pamphlet by Mr. Crutwell of Bath: and yet in the performance before us, we are told, that "Si puerpera infantem lactare nolit, curandum eſt, ut libere fluent lochia; mammas eodem tempore leniter contrahere oportet. *Hoc fit admovendo emplaſtra mammis modicè diſcutientia, quale eſt emplaſtrum commune cum gummi, vel applicatu lini vel lanæ, ſpiritu vini Gallici madefacti, ſub axillis, ipſiſve mammis. Interea autem lac e mammis bis quotidie exſugendum eſt, ne ibi manens obſtructiones pariat.*" We cannot but think that when milk is accumulated in the breaſts, the increaſed irritability that is conſequently induced, renders the ſuction of them extremely painful, and liable to produce inflammation; and that ſuch an operation will rather invite a greater flow of fluid to the gland than unload it; and yet our author contends that ſuch a practice is ſalutary, and that it is oppoſed only by ignorant women, "ignaræ obſtetrices, aliæque puerperæ."

There is no diviſion of the work into chapters or ſections, nor is any particular order ob-

ſerved in the arrangement of the aphoriſms, but a copious index is given at the end of the volume.

